# Radially adjustable Tigertriever demonstrates higher reperfusion compared to self-expanding stent-retrievers during mechanical thrombectomy of large vessel occlusions: a systematic review and meta-analysis

**DOI:** 10.3389/fneur.2026.1839128

**Published:** 2026-07-01

**Authors:** Zain Tariq, Faizan Shahzad, Noor E. Jannat, Tallal Mushtaq Hashmi, Sonesh Amin, Mohammad AlMajali, Qasim Bashir, Jeffrey L. Saver, Besher Shami, Amit Chaudhari

**Affiliations:** 1 Department of Neurointervention, Mercy Health St. Vincent Medical Center, Toledo, OH, United States; 2 Department of Neurology, Rawalpindi Medical University, Rawalpindi, Pakistan; 3 Department of Neurointervention, Dignity Health Mercy Medical Center, Redding, CA, United States; 4 Department of Neuroendovascular Surgery, Ochsner Lafayette General, Lafayette, LA, United States; 5 Department of Neuroendovascular Surgery, Punjab Institute of Neurosciences, Lahore, Pakistan; 6 Department of Interventional Neuroradiology, University of California, Los Angeles, Los Angeles, CA, United States; 7 Department of Neurology, University of Aleppo, Aleppo, Syria

**Keywords:** acute ischemic stroke, large vessel occlusion, mechanical thrombectomy, stent retriever, Tigertriever

## Abstract

**Introduction:**

Mechanical thrombectomy is the standard of care for acute ischemic stroke due to large vessel occlusions. Conventional self-expanding stent retrievers (SE-SRs) deploy with fixed radial force, whereas Tigertriever is a radially adjustable operator-controlled device that allows tailored expansion and modulation of vessel wall pressure. This study systematically compared the efficacy and safety of Tigertriever with SE-SRs in large vessel occlusion thrombectomy.

**Methods:**

A systematic search of PubMed, Cochrane, Embase, and ClinicalTrials.gov was conducted in line with PRISMA guidelines. Eligible studies compared Tigertriever with SE-SRs, with outcomes including rate of successful reperfusion (mTICI ≥ 2b), procedural adverse events (PAEs; composite of procedure-related vasospasm, dissection, perforation, distal emboli, emboli to new territory, and subarachnoid hemorrhage), symptomatic intracranial hemorrhage (sICH) within 24 h post-procedure, functional independence (mRS 0–2) at 90 days and all-cause mortality at 90 days. Odds ratios (OR) with 95% confidence intervals (CI) were pooled using the Mantel–Haenszel method. Risk of bias was assessed using ROBINS-I, and certainty of evidence using the GRADE approach.

**Results:**

Three retrospective cohort studies with 476 patients (210 Tigertriever, 266 SE-SR) were included. Target intracranial vessel occlusion locations were internal carotid artery (ICA), middle cerebral artery (MCA) M1, MCA M2 and basilar artery. Tigertriever achieved significantly higher successful reperfusion compared with SE-SRs [82.8% vs. 77.8%; OR 1.74 (1.07–2.83); *p* = 0.03; *I*^2^ = 0%]. There was a trend towards fewer PAEs, but the difference did not reach statistical significance [17.1% vs. 27.4%; OR 0.72 (0.44–1.16); *p* = 0.17; *I*^2^ = 0%]. Rates of sICH within 24 h, functional independence at 90 days and mortality at 90 days were similar. The overall risk of bias was low to moderate. GRADE-assessed certainty for different outcomes was overall moderate.

**Conclusion:**

In this exploratory meta-analysis of retrospective cohorts, Tigertriever was associated with significantly higher reperfusion rates, while safety and functional outcomes appeared comparable between groups. Findings should be interpreted cautiously given study-level heterogeneity, limited sample size, inconsistent procedural reporting, and the observational nature of included studies.

**Systematic review registration:**

https://www.crd.york.ac.uk/PROSPERO/view/CRD420251161491. The protocol for this meta-analysis was registered on PROSPERO. ID: CRD420251161491.

## Introduction

Mechanical thrombectomy is the standard of care for acute ischemic stroke due to large vessel occlusion, with revascularization typically achieved using aspiration techniques, stent retrievers, or a combination of both (1, 2). Conventional self-expanding stent retrievers (SE-SRs) rely on an intrinsic fixed radial force, which may lead to incomplete clot engagement or increased risk of vessel injury. In contrast, Tigertriever (Rapid Medical, Yokneam, Israel) is a radially adjustable operator-controlled device that allows for incremental expansion under fluoroscopic guidance (3).

Theoretical advantages of radial adjustability include individualized clot-device integration, optimization of wall apposition, and modulation of radial force to potentially reduce endothelial injury or incomplete clot engagement (3). However, these proposed advantages have largely been supported by *in vitro* investigations and early clinical experiences, with limited comparative evidence in real-world patient populations. Importantly, no prior systematic review or meta-analysis has specifically compared radially adjustable stent retrievers with conventional self-expanding stent retrievers.

We hypothesized that this radially adjustable design may improve clot integration while modulating vessel wall interactions. Given the growing interest in device-specific thrombectomy optimization and the potential implications for future stent retriever design, independent evaluation of these proposed benefits in routine clinical practice is warranted. This systematic review and meta-analysis compares Tigertriever with conventional self-expanding stent-retrievers (SE-SRs) in mechanical thrombectomy for large vessel occlusions, focusing on reperfusion efficacy, procedural safety and clinical outcomes.

## Methods

### Registration and search strategy

All data generated or analyzed during this study are included in this article. Further inquiries can be directed to the corresponding author. This meta-analysis was conducted in accordance with the guidelines outlined by the Preferred Reporting Items for Systematic Reviews and Meta-Analyses (4) (Supplementary Table S1). A protocol was developed that clearly defined the objectives, study selection criteria, methods for assessing study quality, clinical and functional stroke outcomes, and statistical procedures. The protocol has been registered on the International Prospective Register of Systematic Reviews (ID: CRD420251161491) to ensure transparency and methodological rigor. As this review did not involve any direct patient data collection or investigation by the authors, ethical approval was not needed.

A comprehensive search of PubMed, Embase, Cochrane Library, and ClinicalTrials.gov was performed through March 2026 without date restrictions. Search terms included Medical Subject Headings (MeSH) and keywords related to acute ischemic stroke, mechanical thrombectomy, and the Tigertriever stent retriever. Reference lists of relevant studies were additionally screened using backward snowballing to identify potentially missed articles. Complete database-specific search strings, search limits, and eligibility filters are provided in the Supplementary materials to facilitate transparency and reproducibility. To further assess reproducibility, the search strategy was independently replicated by two neurointerventionalists, yielding identical study retrieval results.

### Study selection and eligibility criteria

Randomized controlled trials, cohort studies, and case–control studies comparing Tigertriever with conventional self-expanding stent-retrievers were eligible for inclusion. Studies were included if: (1) the population consisted of adults with acute ischemic stroke due to large vessel occlusion undergoing mechanical thrombectomy, (2) outcomes were reported for both Tigertriever and self-expanding stent-retrievers, and (3) at least one predefined outcome was available. These outcomes included successful reperfusion (mTICI ≥2b), procedural adverse events (PAEs; composite of vasospasm, dissection, perforation, distal emboli, emboli to new territory, or subarachnoid hemorrhage), symptomatic intracranial hemorrhage (sICH) within 24 h, functional independence (modified Rankin Scale [mRS] 0–2) at 90 days, and all-cause mortality at 90 days. Case reports, case series, review articles, editorials, in-vitro studies, placebo-controlled studies, studies with no control or historical control, and studies with distal medium vessel occlusions were excluded. Two reviewers (ZT and NEJ) independently screened titles, abstracts, and full texts using the Rayyan software, with disagreements resolved by a third reviewer (JS).

### Data extraction

Data was extracted by two reviewers (TMH and BS) using a standardized collection sheet created in Google Sheets. Extracted variables included study characteristics and patient demographics such as first author, publication year, sample size, mean age, sex distribution, baseline NIHSS, occlusion location, use of intravenous thrombolysis, pre-stroke mRS, onset-to- groin puncture time, and stroke etiology. Where available, procedural variables including first-pass reperfusion, number of thrombectomy passes, and adjunctive aspiration use were also reviewed; however, these data were inconsistently reported across studies and were therefore not amenable to pooled analysis. All discrepancies were resolved by a senior reviewer (QB).

### Risk of bias and quality assessment

Risk of bias for included studies was independently assessed by two reviewers (SA and ZT) using the Cochrane Risk Of Bias In Non-Randomized Studies - of Interventions (ROBINS-I) tool (5). Seven domains were evaluated: confounding, participant selection, intervention classification, deviations from intended interventions, missing data, outcome measurement, and selective reporting. Each domain was graded as low, moderate, serious, or critical risk of bias, with an overall study-level judgment assigned. Because all included studies were retrospective, particular attention was paid to confounding bias, including the presence or absence of propensity-score matching and adjustment methods reported in the source studies. Discrepancies were resolved by consensus with a senior reviewer (MA).

### Outcomes and statistical analysis

The primary efficacy outcome was successful reperfusion, defined as final angiographic reperfusion of the target territory to modified Thrombolysis in Cerebral Infarction ≥2b (mTICI≥2b). The primary safety outcome was procedural adverse events (PAEs), defined as a composite of procedure-related vasospasm, dissection, perforation, distal emboli, emboli to new territory, and subarachnoid hemorrhage.

Secondary outcomes included symptomatic intracranial hemorrhage (sICH) within 24 h, functional independence (mRS 0–2) at 90 days, and all-cause mortality at 90 days. Given study-level variation in sICH definitions (e.g., SITS-MOST, ECASS II, or unspecified criteria), study-specific definitions were recorded and interpreted as a potential source of heterogeneity.

Pooled odds ratios (ORs) with 95% confidence intervals (CIs) were calculated using the Mantel–Haenszel method. Statistical significance was assessed using the Z test. Heterogeneity was evaluated using the *I*^2^ statistic derived from the *χ*^2^ test, with low heterogeneity defined as 25%–50%, moderate as >50%–75%, and substantial as >75% (6). A *p*-value <0.05 was considered statistically significant. All analyses were performed by reviewer FS using Review Manager 5.4.1.

### Certainty of evidence assessment

The certainty of the evidence was evaluated by reviewer ZT using the Grading of Recommendations, Assessment, Development, and Evaluation (GRADE) framework. Based on the GRADE Working Group guidelines, the certainty of the pooled estimates was rated as high, moderate, low, or very low.

### Sensitivity analysis

Sensitivity analyses, including assessments of heterogeneity, were not performed due to the limited number of pooled studies (*n* < 10) and the absence of subgroup data within individual studies. Furthermore, risk of bias due to missing results/publication bias could also not be evaluated using funnel plots or statistical tests. Similarly, subgroup analyses according to stroke etiology, stent retriever subtype, procedural approach, or timing variables were not feasible because of limited study availability and lack of patient-level granularity.

## Results

### Search results

**Figure 1:**
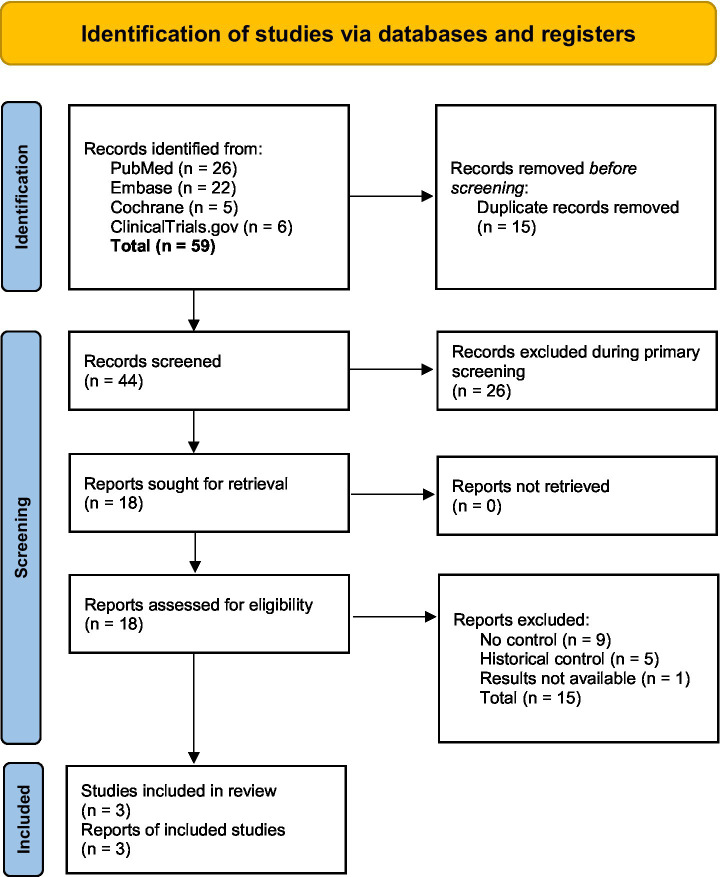
PRISMA Flowchart showing the screening process.

The systematic search identified 59 records. After removal of 15 duplicates, 44 titles and abstracts were screened, of which 36 were excluded. Full texts of the remaining 18 articles were assessed, and 15 were excluded during secondary screening. Three studies met the inclusion criteria and were included in the final analysis; all were retrospective cohort studies. No randomized controlled studies directly comparing Tigertriever with conventional self-expanding stent retrievers were identified. The study selection process is summarized in Figure 1.

### Baseline characteristics

The three included studies comprised 476 patients: 210 treated with the Tigertriever stent retriever and 266 treated with conventional self-expanding stent-retrievers. Mean age ranged from 66.7–81.1 years in the Tigertriever group and 66.8–79.2 years in the comparator group. Males represented 100 patients (47.6%) in the Tigertriever group and 137 patients (51.5%) in the self-expanding stent-retriever group. Baseline variables including stroke etiology, onset-to-groin puncture time, and procedural characteristics were variably reported across studies and were therefore not amenable to pooled adjusted analyses. Detailed patient baseline characteristics are summarized for each of the studies in Table 1.

**Table 1:** Baseline characteristics of individual studies.

Study characteristics	Study groups	Study author and year		
		Vu et al. (2025) (7)	Shindo et al. (2025) (8)	Piasecki et al. (2023) (9)
Study design		Retrospective single-center cohort study (propensity-score matched)	Retrospective single-center cohort study	Retrospective single- center cohort study (propensity-score matched)
SE-SR used		Solitaire X (Medtronic, USA)	Solitaire, EmboTrap (Johnson & Johnson MedTech, USA), Trevo (Stryker, USA), Tron (JIMRO, Japan)	Solitaire X, Trevo, pRESET (Phenox, Germany)
Sample size, *n*	Tigertriever	134	24	52
	SE-SR	134	80	52
Age (years), mean (SD)	Tigertriever	66.7 (13.4)	81.1 (10.3)	68
	SE-SR	66.8 (12.4)	79.2 (12.8)	71
Gender males, n (%)	Tigertriever	64 (47.8)	11 (45.8)	25 (51.0)
	SE-SR	77 (57.5)	36 (45.0)	24 (49.0)
NIHSS on admission, median (IQR)	Tigertriever	14 (11–15)	17 (8–25)	16
	SE-SR	12.5 (10–16)	16 (9–24)	15
Time from onset to groin puncture (minutes), median (IQR)	Tigertriever	327.5 (235.8–350)	411 (304–644)	247
	SE-SR	214 (175.8–270)	370 (237–596)	228
Rate of IV thrombolysis, *n* (%)	Tigertriever	40 (30.1)	5^a^ (20.8)	35 (58.0)
	SE-SR	53 (39.2)	18^a^ (22.5)	55 (69.0)
Pre-stroke mRS, *n* (%)				
0–1	Tigertriever	NR^b^	10 (41.7)	46 (88.5)
	SE-SR	NR^b^	45 (56.3)	44 (84.6)
>1	Tigertriever	NR^b^	14 (58.3)	6 (11.5)
	SE-SR	NR^b^	35 (43.8)	8 (15.4)
Site of occlusion, *n* (%)				
Intracranial ICA	Tigertriever	10 (7.4)	5 (20.8)	13 (15.0)
	SE-SR	10 (7.4)	16 (20.0)	13 (15.0)
MCA M1	Tigertriever	61 (45.6)	8 (33.3)	22 (46.0)
	SE-SR	61 (45.6)	36 (45.0)	26 (50.0)
Both intracranial ICA and M1	Tigertriever	34 (25.4)	NR	NR
	SE-SR	34 (25.4)	NR	NR
MCA M2	Tigertriever	16 (11.9)	11 (45.8)	14 (17.0)
	SE-SR	16 (11.9)	28 (35.0)	5 (10.0)
Basilar artery	Tigertriever	13 (9.7)	0 (0.0)	2 (4.0)
	SE-SR	13 (9.7)	0 (0.0)	3 (6.0)
Tandem occlusion	Tigertriever	NR	2 (10.4)	1 (2.0)
	SE-SR	NR	8 (11.1)	5 (10.0)
Etiology of LVO, *n* (%)				
Embolic (cardiogenic/non-cardiogenic)	Tigertriever	85 (63.4)	19 (79.2)	NR
	SE-SR	68 (50.7)	71 (88.8)	NR
ICAD	Tigertriever	45 (33.6)	5 (20.8)	NR
	SE-SR	57 (42.5)	9 (11.2)	NR

ICA, internal carotid artery; ICAD, intracranial atherosclerotic disease; IQR, interquartile range; IV, intravenous; LVO, large vessel occlusion; MCA, middle cerebral artery; mRS, modified Rankin Scale; NIHSS, National Institutes of Health Stroke Scale; NR, not reported; SE-SR, self-expanding stent retriever.

a This study was conducted in Japan. Hence, subjects received a lower dose of tPA (0.6 mg/kg).

b This study excluded subjects with pre-stroke mRS >2 but exact proportions of mRS 0–1 versus mRS 2 are not reported.

## Outcomes

### Successful reperfusion (mTICI ≥2b)

**Figure 2:**
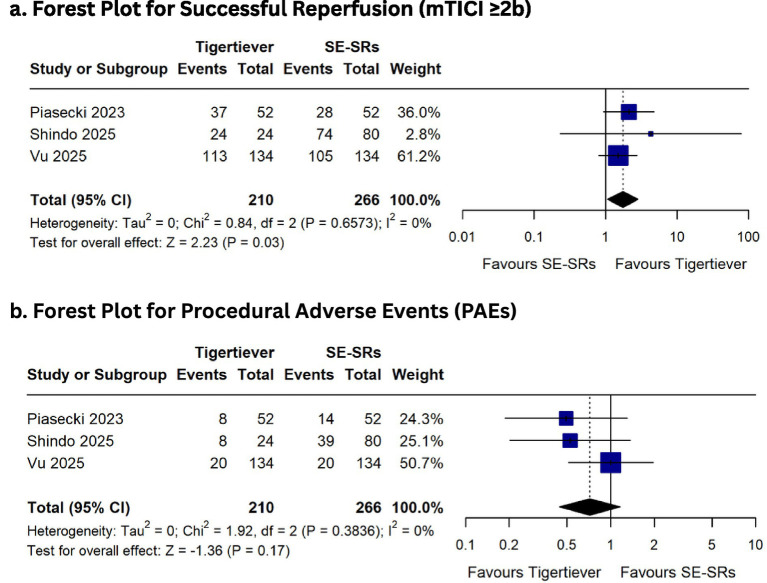
Forest plots for successful reperfusion and procedural adverse events (primary efficacy and safety endpoints, respectively).

All studies reported successful reperfusion. Rates were higher in the Tigertriever group compared with self-expanding stent-retrievers [174/210 (82.8%) vs. 207/266 (77.8%)]. Pooled analysis demonstrated significantly higher reperfusion with Tigertriever [OR 1.74 (95% CI 1.07–2.83); *p* = 0.03], with no observed heterogeneity (*I*^2^ = 0%). These findings suggest a procedural association with improved angiographic reperfusion; however, given the observational nature of included studies, causality cannot be inferred. Results are shown in Figure 2a.

### Procedural adverse events (PAEs)

All studies reported procedural adverse events, defined as a composite of procedure-related vasospasm, dissection, perforation, distal emboli, emboli to new territory, or subarachnoid hemorrhage. There were numerically fewer events in the Tigertriever group (36/210; 17.1%) compared with self-expanding stent-retrievers (73/266; 27.4%), although this did not reach statistical significance [OR 0.72 (95% CI 0.44–1.16); *p* = 0.17]. No heterogeneity was observed (*I*^2^ = 0%). Because first-pass effect, number of thrombectomy passes, rescue techniques, and adjunctive aspiration use were inconsistently reported, procedural efficacy should be interpreted cautiously. Results are shown in Figure 2b.

### sICH within 24 h post-procedure

**Figure 3:**
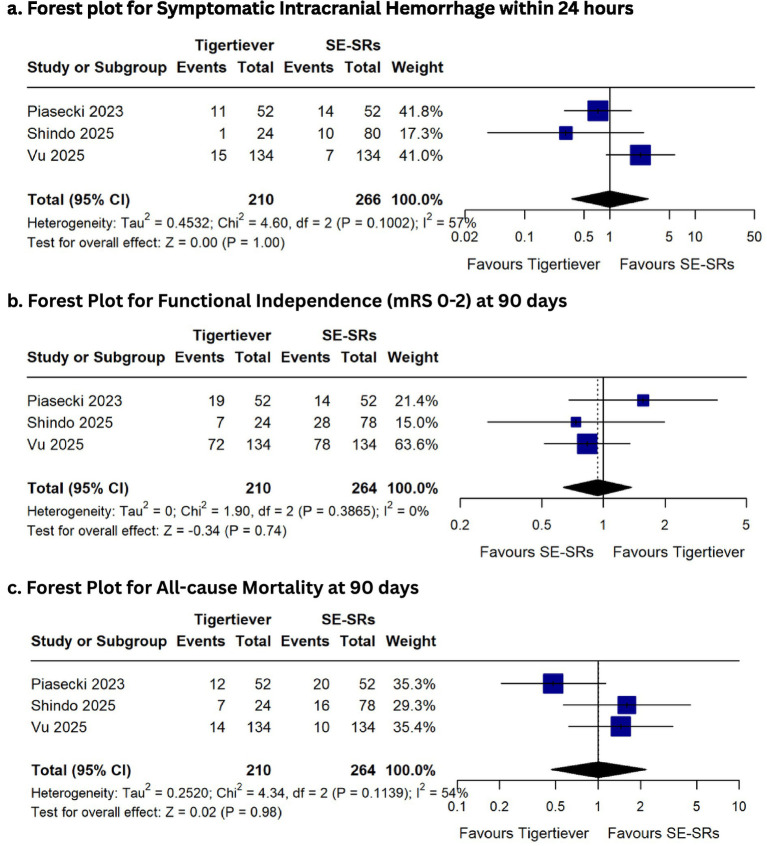
Forest plots for secondary outcomes, including symptomatic intracranial hemorrhage within 24 h, functional independence (mRS 0–2) at 90 days, and all-cause mortality at 90 days.

All studies reported symptomatic intracranial hemorrhage (sICH), although definitions varied. Vu et al. (7) applied the SITS-MOST criteria within 24 h, Shindo et al. (8) used the ECASS II criteria within 22–36 h, and Piasecki et al. (9) reported sICH within 24 h without specifying criteria. The pooled rate of sICH was similar between groups [27/210 (12.9%) vs. 31/266 (11.7%)] [OR 1.00 (95% CI 0.36–2.81); *p* = 1.00], with moderate heterogeneity (*I*^2^ = 57%). Variation in sICH definitions across studies represents a potential source of outcome heterogeneity and should be considered when interpreting pooled estimates. Results are shown in Figure 3a.

### Functional independence (mRS 0–2) at 90 days

All studies reported functional independence at 90 days. Rates of functional independence were similar between groups [98/210 (46.7%) vs. 120/266 (45.1%)] [OR 0.94 (95% CI 0.64–1.38); p. 0.74]. No heterogeneity was observed (*I*^2^ = 0%). Despite differences in reperfusion, no measurable difference in functional independence was observed, although interpretation is limited by confounding factors not captured in pooled study-level analyses. Results are shown in Figure 3b.

### All-cause mortality at 90 days

All three studies reported 90-day mortality. Rates were comparable between groups [33/210 (15.7%) vs. 46/266 (17.3%)] [OR 1.01 (95% CI 0.47–2.19); *p* = 0.98], with moderate heterogeneity across studies (I^2^ = 54%). Results are shown in Figure 3c.

### Risk of bias and quality assessment

**Figure 4:**
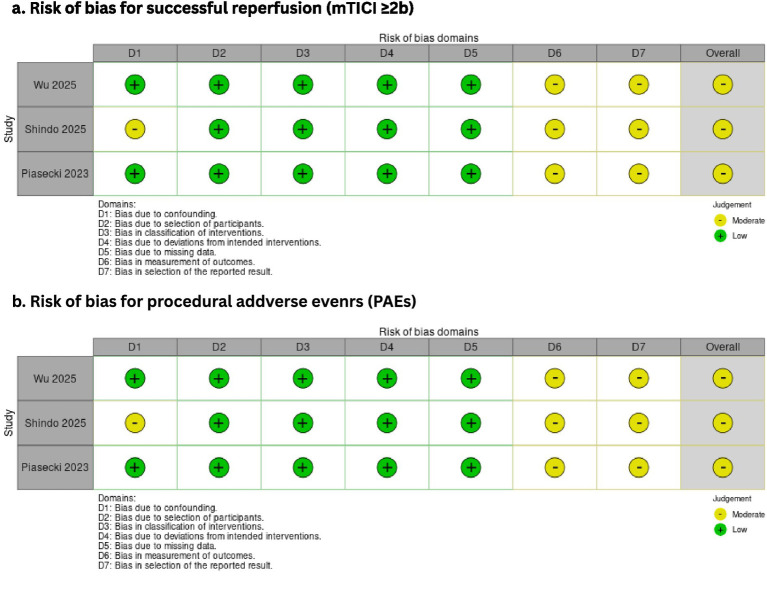
Traffic plot showing the risk of bias in included studies.

Risk of bias was assessed using the ROBINS-I tool. Overall, studies demonstrated low risk of bias across most domains, moderate concerns related primarily to confounding inherent to retrospective study design and selective reporting. Two studies incorporated propensity-score matching to reduce measured confounding, whereas one retrospective cohort lacked adjustment methods and was therefore judged to have greater confounding risk. Participant selection, classification of interventions, deviations from intended interventions, missing data, and outcome measurement were judged to have low risk. The traffic plot summarizing risk of bias for primary outcomes is presented in Figure 4, with additional plots for secondary outcomes in Supplementary Figures S1–S3.

### Certainty of evidence

Certainty of evidence was evaluated using the GRADE approach. Successful reperfusion, procedural adverse events, and good functional outcome were rated as moderate-certainty evidence based on consistent results and acceptable precision despite the observational study design. Symptomatic intracranial hemorrhage (*I*^2^ = 57%) and 90-day mortality (*I*^2^ = 54%) were graded as low-certainty evidence due to moderate heterogeneity. The certainty of pooled estimates should be interpreted within the context of retrospective study design, limited sample size, study-level heterogeneity, and lack of patient-level adjustment. Overall certainty ranged from moderate to low. The summary of findings and GRADE assessment are presented in Supplementary Table S4.

## Discussion

This systematic review and meta-analysis pooled comparative retrospective data evaluating the radially adjustable Tigertriever stent retriever versus conventional self-expanding stent-retrievers (SE-SRs) for mechanical thrombectomy in large vessel occlusion stroke. Tigertriever demonstrated significantly higher rates of successful reperfusion (mTICI ≥2b) compared with conventional devices. There were also numerically fewer procedural adverse events, although this difference did not reach statistical significance. Rates of symptomatic intracranial hemorrhage within 24 h, functional independence at 90 days, and all-cause mortality at 90 days were similar between the two device groups. Taken together, these findings suggest a potential procedural advantage in angiographic reperfusion associated with radially adjustable stent retrievers, while observed safety and functional outcomes remained broadly comparable. Given the retrospective nature and limited size of the available literature, findings should be considered exploratory and hypothesis-generating.

These findings are consonant with and importantly extend prior studies. As a meta-analysis, the results are perforce consistent with the component included investigations. They also accord with the finding in the registration trial of the Tigertriever device of higher reperfusion rates and favorable safety outcomes, compared with historical performance benchmarks derived from prior trials of SE-SRs (3). However, the registration study’s single-arm design limited direct comparative evidence and was performed in a highly selected clinical trial population. Importantly, our study represents, to our knowledge, the first systematic review and meta-analysis specifically comparing a radially adjustable stent retriever against conventional self-expanding platforms, thereby addressing an evidence gap regarding whether proposed technical advantages translate into comparative real-world procedural outcomes.

Our systematic review and meta-analysis is the first to specifically compare Tigertriever against a variety of SE-SRs for large vessel occlusion thrombectomy. In contrast to the TIGER trial that compared outcomes with historical benchmarks from clinical trials that used only Solitaire or Trevo stent retrievers, our comparison additionally included studies evaluating Embotrap, preSET and TRON (all of which are FDA-approved, except the last which is approved in Japan), reflecting real-world device utilization. In addition, the proportion of M2 and basilar occlusions in our meta-analysis ranged from 10% to 46% (vs. 23% in the TIGER trial) and 0%–10% (vs. 3% in the TIGER trial), respectively, and two of the studies enrolled up to 11% of subjects with tandem occlusions. While this increases clinical generalizability, it also introduces device-level and anatomical heterogeneity within the comparator arm and therefore represents an important limitation of pooled interpretation. Device-specific subgroup analyses were not feasible because only three comparative retrospective studies met inclusion criteria. Overall, this study design closely reflects real-world comparative performance between radially adjustable and conventional stent-retriever platforms.

Tigertriever was associated with significantly higher rates of successful reperfusion (mTICI ≥2b) compared with conventional SE-SRs. Although one plausible explanation may relate to the device’s radially adjustable design, allowing individualized expansion and theoretically improved clot-device integration and vessel wall apposition, such mechanistic explanations remain speculative and are not directly testable within the included clinical studies. Accordingly, these findings should not be interpreted as mechanistic proof, but rather as clinical observations that may motivate future dedicated mechanistic and translational investigations.

Importantly, despite theoretical concerns that operator-controlled expansion or the distal delivery wire rigidity in radially adjustable devices could increase risk of vessel injury, rates of symptomatic intracranial hemorrhage were similar between groups, suggesting comparable procedural safety. Notably, it remains underexplored why the higher reperfusion rates did not translate into improved functional outcomes or mortality at 90 days. Functional recovery after stroke is influenced by numerous downstream variables, including infarct core volume, collateral status, baseline disability, age, blood pressure management, post-thrombectomy complications, rehabilitation access, and time to reperfusion, that were not amenable to patient-level adjustment in the present study. Accordingly, the current analysis was designed primarily to evaluate procedural and angiographic performance rather than establish causal relationships between stent retriever type and long-term functional independence.

Several limitations warrant emphasis. First, only three retrospective studies met inclusion criteria, limiting statistical power and precluding formal publication bias assessment. Second, all included studies were retrospective and lacked access to patient-level data, preventing adjustment for important confounders such as stroke etiology, onset-to-groin puncture time, infarct characteristics, or post-procedural care variables. Third, pooling multiple self-expanding stent retrievers into a single comparator group introduces device-level heterogeneity. Fourth, symptomatic intracranial hemorrhage definitions varied across studies (SITS-MOST, ECASS II, or unspecified definitions), introducing additional outcome heterogeneity. Fifth, key procedural variables—including first-pass effect, number of thrombectomy attempts, adjunctive aspiration techniques, rescue strategies, and per-pass device utilization—were inconsistently reported and therefore could not be pooled. Finally, while two included studies used propensity-score matching to reduce measured confounding, residual unmeasured confounding remains unavoidable in retrospective comparative analyses.

Despite these limitations, this systematic review and meta-analysis addresses an important unmet comparative question in thrombectomy device evaluation by independently assessing whether the proposed technical distinction of radial adjustability is associated with measurable procedural differences in real-world practice. While prospective comparative studies are needed to confirm these findings, the ability to tailor radial expansion during clot retrieval represents a meaningful technical distinction that may offer advantages in selected anatomical and pathological scenarios. While prospective comparative studies are needed to validate these findings, the concept of tailored radial expansion during clot retrieval represents a potentially important avenue for future thrombectomy device development. Future prospective studies and patient-level pooled analyses will be essential to identify which clot compositions, vascular anatomies, stroke etiologies (e.g., intracranial atherosclerotic disease versus cardioembolism), and procedural contexts may derive the greatest benefit from radially adjustable platforms.

## Conclusion

In this exploratory systematic review and meta-analysis of retrospective comparative studies, Tigertriever was associated with significantly higher reperfusion rates than self-expanding stent-retrievers, whereas procedural safety, functional independence, and mortality appeared comparable between groups. These findings should be interpreted cautiously given the limited number of studies, retrospective study design, heterogeneity in comparator devices and hemorrhage definitions, inconsistent reporting of key procedural variables, and inability to adjust for patient-level confounding. Further prospective comparative studies are warranted to validate these findings and clarify the role of radially adjustable stent retrievers in mechanical thrombectomy.

## Data Availability

The original contributions presented in the study are included in the article/Supplementary material, further inquiries can be directed to the corresponding author.
