# Correction: Radially adjustable Tigertriever demonstrates higher reperfusion compared to self-expanding stent-retrievers during mechanical thrombectomy of large vessel occlusions: a systematic review and meta-analysis

**DOI:** 10.3389/fneur.2026.1939142

**Published:** 2026-07-29

**Authors:** Zain Tariq, Faizan Shahzad, Noor E Jannat, Tallal Mushtaq Hashmi, Sonesh Amin, Mohammad AlMajali, Qasim Bashir, Jeffrey L. Saver, Besher Shami, Amit Chaudhari

**Affiliations:** 1 Department of Neurointervention, Mercy Health St. Vincent Medical Center, Toledo, OH, United States; 2 Department of Neurology, Rawalpindi Medical University, Rawalpindi, Pakistan; 3 Department of Neurointervention, Dignity Health Mercy Medical Center, Redding, CA, United States; 4 Department of Neuroendovascular Surgery, Ochsner Lafayette General, Lafayette, LA, United States; 5 Department of Neuroendovascular Surgery, Punjab Institute of Neurosciences, Lahore, Pakistan; 6 Department of Interventional Neuroradiology, University of California, Los Angeles, Los Angeles, CA, United States; 7 Department of Neurology, University of Aleppo, Aleppo, Syria

**Keywords:** acute ischemic stroke, large vessel occlusion, mechanical thrombectomy, stent retriever, Tigertriever

The funder Rapid Medical was erroneously omitted.

The corrected **Funding** statement is:

“The author(s) declared that financial support was received for this work and/or its publication. The Article Processing Charge (APC) for this publication was covered by Rapid Medical. Rapid Medical had no role in the study design, conduct, analysis, interpretation of the data, manuscript preparation, or the decision to submit the manuscript for publication.”

The **Conflict of interest** statement was erroneously given as: “The author(s) declared that this work was conducted in the absence of any commercial or financial relationships that could be construed as a potential conflict of interest.”

The correct **Conflict of interest** statement is:

“AC and JS serve as consultants for Rapid Medical. They received no funding or financial support for this project from Rapid Medical other than coverage of the Article Processing Charge (APC).

The remaining author(s) declared that this work was conducted in the absence of any commercial or financial relationships that could be construed as a potential conflict of interest.”

The original version of this article has been updated.

