# Supplementary material for: Radially adjustable Tigertriever demonstrates higher reperfusion compared to self-expanding stent-retrievers during mechanical thrombectomy of large vessel occlusions: a systematic review and meta-analysis
Source: Front Neurol. 2026 Jul 1;17:1839128. doi: 10.3389/fneur.2026.1839128 (PMC13368976; doi:10.3389/fneur.2026.1839128)

**Radially Adjustable Tigertriever Demonstrates Higher Reperfusion Compared to Self-expanding Stent-retrievers During Mechanical Thrombectomy of Large Vessel Occlusions: A Systematic Review and Meta-analysis**

Zain Tariq, MD^1^; Faizan Shahzad, MD^2^; Noor E Jannat, MBBS^2^; Tallal Mushtaq Hashmi, MBBS^2^; Sonesh Amin, MD^3^; Mohammad AlMajali, MD^4^; Qasim Bashir, MD^5^; Jeffrey L. Saver^6^; Besher Shami, MD^7^; Amit Chaudhari, MD, PhD^1,3^

**Affiliations:**

1. Department of Neurointervention, Mercy Health St. Vincent Medical Center, Toledo, OH. United States of America.
2. Department of Neurology, Rawalpindi Medical University, Rawalpindi. Pakistan.
3. Department of Neurointervention, Dignity Health Mercy Medical Center, Redding, CA, United States of America
4. Department of Neuroendovascular Surgery, Ochsner Lafayette General, Lafayette, LA. United States of America.
5. Department of Neuroendovascular Surgery, Punjab Institute of Neurosciences, Lahore. Pakistan.
6. Department of Interventional Neuroradiology, University of California, Los Angeles. United States of America.
7. University of Aleppo, Aleppo. Syria.

**Corresponding author:**

Name: Amit Chaudhari, MD, PhD

Email: [amitchaudharimd@gmail.com](mailto:amitchaudharimd@gmail.com)

**Table S1. PRISMA Checklist**

| **Section and Topic** | **Item #** | **Checklist item** | **Location where item is reported** |
| --- | --- | --- | --- |
| **TITLE** | | |  |
| Title | 1 | Identify the report as a systematic review. | ✔ |
| **ABSTRACT** | | |  |
| Abstract | 2 | See the PRISMA 2020 for Abstracts checklist. | ✔ |
| **INTRODUCTION** | | |  |
| Rationale | 3 | Describe the rationale for the review in the context of existing knowledge. | ✔ |
| Objectives | 4 | Provide an explicit statement of the objective(s) or question(s) the review addresses. | ✔ |
| **METHODS** | | |  |
| Eligibility criteria | 5 | Specify the inclusion and exclusion criteria for the review and how studies were grouped for the syntheses. | ✔ |
| Information sources | 6 | Specify all databases, registers, websites, organisations, reference lists and other sources searched or consulted to identify studies. Specify the date when each source was last searched or consulted. | ✔ |
| Search strategy | 7 | Present the full search strategies for all databases, registers and websites, including any filters and limits used. | Table S2 |
| Selection process | 8 | Specify the methods used to decide whether a study met the inclusion criteria of the review, including how many reviewers screened each record and each report retrieved, whether they worked independently, and if applicable, details of automation tools used in the process. | ✔ |
| Data collection process | 9 | Specify the methods used to collect data from reports, including how many reviewers collected data from each report, whether they worked independently, any processes for obtaining or confirming data from study investigators, and if applicable, details of automation tools used in the process. | ✔ |
| Data items | 10a | List and define all outcomes for which data were sought. Specify whether all results that were compatible with each outcome domain in each study were sought (e.g. for all measures, time points, analyses), and if not, the methods used to decide which results to collect. | ✔ |
|  | 10b | List and define all other variables for which data were sought (e.g. participant and intervention characteristics, funding sources). Describe any assumptions made about any missing or unclear information. | ✔ |
| Study risk of bias assessment | 11 | Specify the methods used to assess risk of bias in the included studies, including details of the tool(s) used, how many reviewers assessed each study and whether they worked independently, and if applicable, details of automation tools used in the process. | ✔ |
| Effect measures | 12 | Specify for each outcome the effect measure(s) (e.g. risk ratio, mean difference) used in the synthesis or presentation of results. | ✔ |
| Synthesis methods | 13a | Describe the processes used to decide which studies were eligible for each synthesis (e.g. tabulating the study intervention characteristics and comparing against the planned groups for each synthesis (item #5)). | ✔ |
|  | 13b | Describe any methods required to prepare the data for presentation or synthesis, such as handling of missing summary statistics, or data conversions. | ✔ |
|  | 13c | Describe any methods used to tabulate or visually display results of individual studies and syntheses. | ✔ |
|  | 13d | Describe any methods used to synthesize results and provide a rationale for the choice(s). If meta-analysis was performed, describe the model(s), method(s) to identify the presence and extent of statistical heterogeneity, and software package(s) used. | ✔ |
|  | 13e | Describe any methods used to explore possible causes of heterogeneity among study results (e.g. subgroup analysis, meta-regression). | ✔ |
|  | 13f | Describe any sensitivity analyses conducted to assess robustness of the synthesized results. | ✔ |
| Reporting bias assessment | 14 | Describe any methods used to assess risk of bias due to missing results in a synthesis (arising from reporting biases). | ✔ |
| Certainty assessment | 15 | Describe any methods used to assess certainty (or confidence) in the body of evidence for an outcome. | ✔ |
| **RESULTS** | | |  |
| Study selection | 16a | Describe the results of the search and selection process, from the number of records identified in the search to the number of studies included in the review, ideally using a flow diagram. | Figure 1 |
|  | 16b | Cite studies that might appear to meet the inclusion criteria, but which were excluded, and explain why they were excluded. | NA |
| Study characteristics | 17 | Cite each included study and present its characteristics. | Table 1 |
| Risk of bias in studies | 18 | Present assessments of risk of bias for each included study. | Figures 4, S1-S3 |
| Results of individual studies | 19 | For all outcomes, present, for each study: (a) summary statistics for each group (where appropriate) and (b) an effect estimate and its precision (e.g. confidence/credible interval), ideally using structured tables or plots. | Figures 2, 3 |
| Results of syntheses | 20a | For each synthesis, briefly summarise the characteristics and risk of bias among contributing studies. | ✔ |
|  | 20b | Present results of all statistical syntheses conducted. If meta-analysis was done, present for each the summary estimate and its precision (e.g. confidence/credible interval) and measures of statistical heterogeneity. If comparing groups, describe the direction of the effect. | ✔ |
|  | 20c | Present results of all investigations of possible causes of heterogeneity among study results. | NA (see item 13e) |
|  | 20d | Present results of all sensitivity analyses conducted to assess the robustness of the synthesized results. | NA (see item 13f) |
| Reporting biases | 21 | Present assessments of risk of bias due to missing results (arising from reporting biases) for each synthesis assessed. | ✔ |
| Certainty of evidence | 22 | Present assessments of certainty (or confidence) in the body of evidence for each outcome assessed. | ✔ |
| **DISCUSSION** | | |  |
| Discussion | 23a | Provide a general interpretation of the results in the context of other evidence. | ✔ |
|  | 23b | Discuss any limitations of the evidence included in the review. | ✔ |
|  | 23c | Discuss any limitations of the review processes used. | ✔ |
|  | 23d | Discuss implications of the results for practice, policy, and future research. | ✔ |
| **OTHER INFORMATION** | | |  |
| Registration and protocol | 24a | Provide registration information for the review, including register name and registration number, or state that the review was not registered. | ✔ |
|  | 24b | Indicate where the review protocol can be accessed, or state that a protocol was not prepared. | ✔ |
|  | 24c | Describe and explain any amendments to information provided at registration or in the protocol. | ✔ |
| Support | 25 | Describe sources of financial or non-financial support for the review, and the role of the funders or sponsors in the review. | ✔ |
| Competing interests | 26 | Declare any competing interests of review authors. | ✔ |
| Availability of data, code and other materials | 27 | Report which of the following are publicly available and where they can be found: template data collection forms; data extracted from included studies; data used for all analyses; analytic code; any other materials used in the review. | ✔ |

**Table S2. PICOS Framework**

| Population (P) | Patients with acute ischemic stroke (AIS) due to large vessel occlusion (LVO) |
| --- | --- |
| Intervention (I) | Tigertriever (radially adjustable stent-retriever) |
| Comparators (C) | Conventional self-expanding stent-retrievers |
| Outcome (O) | Primary:   - Successful reperfusion (mTICI ≥2b) - Procedural adverse events (composite of procedure-related vasospasm, dissection, perforation, distal emboli, emboli to new territory, and subarachnoid hemorrhage)   Secondary:   - Symptomatic intracranial hemorrhage (sICH) within 24 hours - Functional independence (mRS 0-2) at 90 days - All-cause mortality at 90 days |
| Study (S) | - Randomized controlled trials - Cohort studies - Case control studies |

**Table S3. Search Strategy**

| **Database** | **Search String** | **Filters** | **Records** |
| --- | --- | --- | --- |
| PubMED | ((((((((((Tigertriever) OR (Tigertriever 13)) OR (Tigertriever 17)) OR (Tigertriever 21)) OR (Adjustable stent-retriever)) OR (Adjustable stent retriever)) OR (Adjustable stentriever)) OR (Radially adjustable stent-retriever)) OR (Radially adjustable stent retriever)) OR (Radially adjustable stentriever)) AND (((((((((((((((((((((((((((Ischemic Stroke) OR (Ischemic Strokes)) OR (Stroke, Ischemic)) OR (Ischaemic Stroke)) OR (Ischaemic Strokes)) OR (Stroke, Ischaemic)) OR (Acute Ischemic Stroke)) OR (Acute Ischemic Strokes)) OR (Ischemic Stroke, Acute)) OR (Stroke, Acute Ischemic)) OR (Cryptogenic Ischemic Stroke)) OR (Cryptogenic Ischemic Strokes)) OR (Ischemic Stroke, Cryptogenic)) OR (Stroke, Cryptogenic Ischemic)) OR (Cryptogenic Embolism Stroke)) OR (Cryptogenic Embolism Strokes)) OR (Embolism Stroke, Cryptogenic)) OR (Stroke, Cryptogenic Embolism)) OR (Cryptogenic Stroke)) OR (Cryptogenic Strokes)) OR (Stroke, Cryptogenic)) OR (Wake-up Stroke)) OR (Stroke, Wake-up)) OR (Wake up Stroke)) OR (Wake-up Strokes)) OR (Large vessel occlusion)) OR (Large-vessel occlusion)) | None | 26 |
| Embase | ('ischemic stroke'/exp OR 'ischaemic stroke' OR 'ischemic stroke' OR 'acute ischemic stroke'/exp OR 'acute ischaemic stroke' OR 'acute ischemic stroke' OR 'cerebrovascular accident'/exp OR 'cva' OR 'accident, cerebrovascular' OR 'acute cerebrovascular lesion' OR 'acute focal cerebral vasculopathy' OR 'acute stroke' OR 'apoplectic stroke' OR 'apoplexia' OR 'apoplexy' OR 'blood flow disturbance, brain' OR 'brain accident' OR 'brain attack' OR 'brain blood flow disturbance' OR 'brain insult' OR 'brain insultus' OR 'brain vascular accident' OR 'cerebral apoplexia' OR 'cerebral insult' OR 'cerebral stroke' OR 'cerebral vascular accident' OR 'cerebral vascular insufficiency' OR 'cerebro vascular accident' OR 'cerebrovascular accident' OR 'cerebrovascular arrest' OR 'cerebrovascular failure' OR 'cerebrovascular injury' OR 'cerebrovascular insufficiency' OR 'cerebrovascular insult' OR 'cerebrum vascular accident' OR 'cryptogenic stroke' OR 'insultus cerebralis' OR 'ischaemic seizure' OR 'ischemic seizure' OR 'stroke' OR 'thrombotic stroke' OR 'cardioembolic stroke'/exp OR 'cardioembolic stroke' OR 'embolic stroke' OR 'wake up stroke'/exp OR 'awakening stroke' OR 'stroke at awakening' OR 'stroke at wake up' OR 'wake up stroke' OR 'wakeup stroke' OR 'large vessel occlusion'/exp) AND ('tigertriever'/exp OR 'adjustable stent retriever' OR 'adjustable stent-retriever' OR 'adjustable stentriever' OR 'radially adjustable stent- retriever' OR 'radially adjustable stent retriever' OR 'radially adjustable stentriever') | None | 22 |
| Cochrane Library | ((((((((((Tigertriever) OR (Tigertriever 13)) OR (Tigertriever 17)) OR (Tigertriever 21)) OR (Adjustable stent-retriever)) OR (Adjustable stent retriever)) OR (Adjustable stentriever)) OR (Radially adjustable stent-retriever)) OR (Radially adjustable stent retriever)) OR (Radially adjustable stentriever)) AND (((((((((((((((((((((((((((Ischemic Stroke) OR (Ischemic Strokes)) OR (Stroke, Ischemic)) OR (Ischaemic Stroke)) OR (Ischaemic Strokes)) OR (Stroke, Ischaemic)) OR (Acute Ischemic Stroke)) OR (Acute Ischemic Strokes)) OR (Ischemic Stroke, Acute)) OR (Stroke, Acute Ischemic)) OR (Cryptogenic Ischemic Stroke)) OR (Cryptogenic Ischemic Strokes)) OR (Ischemic Stroke, Cryptogenic)) OR (Stroke, Cryptogenic Ischemic)) OR (Cryptogenic Embolism Stroke)) OR (Cryptogenic Embolism Strokes)) OR (Embolism Stroke, Cryptogenic)) OR (Stroke, Cryptogenic Embolism)) OR (Cryptogenic Stroke)) OR (Cryptogenic Strokes)) OR (Stroke, Cryptogenic)) OR (Wake-up Stroke)) OR (Stroke, Wake-up)) OR (Wake up Stroke)) OR (Wake-up Strokes)) OR (Large vessel occlusion)) OR (Large-vessel occlusion)) | None | 5 |
| ClinicalTrials.gov | ((((((((((Tigertriever) OR (Tigertriever 13)) OR (Tigertriever 17)) OR (Tigertriever 21)) OR (Adjustable stent-retriever)) OR (Adjustable stent retriever)) OR (Adjustable stentriever)) OR (Radially adjustable stent-retriever)) OR (Radially adjustable stent retriever)) OR (Radially adjustable stentriever)) AND (((((((((((((((((((((((((((Ischemic Stroke) OR (Ischemic Strokes)) OR (Stroke, Ischemic)) OR (Ischaemic Stroke)) OR (Ischaemic Strokes)) OR (Stroke, Ischaemic)) OR (Acute Ischemic Stroke)) OR (Acute Ischemic Strokes)) OR (Ischemic Stroke, Acute)) OR (Stroke, Acute Ischemic)) OR (Cryptogenic Ischemic Stroke)) OR (Cryptogenic Ischemic Strokes)) OR (Ischemic Stroke, Cryptogenic)) OR (Stroke, Cryptogenic Ischemic)) OR (Cryptogenic Embolism Stroke)) OR (Cryptogenic Embolism Strokes)) OR (Embolism Stroke, Cryptogenic)) OR (Stroke, Cryptogenic Embolism)) OR (Cryptogenic Stroke)) OR (Cryptogenic Strokes)) OR (Stroke, Cryptogenic)) OR (Wake-up Stroke)) OR (Stroke, Wake-up)) OR (Wake up Stroke)) OR (Wake-up Strokes)) OR (Large vessel occlusion)) OR (Large-vessel occlusion)) | None | 6 |

**Table S4. Summary of findings table for GRADE assessment**

| Outcomes | **Anticipated absolute effects^*^** (95% CI) | | Relative effect (95% CI) | № of participants (studies) | Certainty of the evidence (GRADE) |
| --- | --- | --- | --- | --- | --- |
|  | **Risk with SE-SR** | **Risk with Tigertriever** |  |  |  |
| Successful reperfusion | 78 per 100 | **83 per 100** | **OR 1.74** (1.07 to 2.83) | 476 (3 retrospective cohort studies) | ⨁⨁⨁◯  Moderate^a^ |
| Procedural adverse events (PAEs) | 27 per 100 | **17 per 100** | **OR 0.77** (0.48 to 1.25) | 476 (3 retrospective cohort studies) | ⨁⨁⨁◯  Moderate^a^ |
| Symptomatic intracranial hemorrhage (sICH) within 24 hours | 12 per 100 | **13 per 100** | **OR 1.00** (0.64 to 1.38) | 476 (3 retrospective cohort studies) | ⨁⨁◯◯ Low^b^ |
| Functional independence (mRS 0-2) at 90 days | 45 per 100 | **47 per 100** | **OR 0.94** (0.64 to 1.38) | 476 (3 retrospective cohort studies) | ⨁⨁⨁◯  Moderate^a^ |
| All-cause mortality at 90 days | 17 per 100 | **16 per 100** | **OR 1.01** (0.47 to 2.19) | 476 (3 retrospective cohort studies) | ⨁⨁◯◯ Low^b^ |

**GRADE Working Group grades of evidence**
**High certainty:** we are very confident that the true effect lies close to that of the estimate of the effect.
**Moderate certainty:** we are moderately confident in the effect estimate: the true effect is likely to be close to the estimate of the effect, but there is a possibility that it is substantially different.
**Low certainty:** our confidence in the effect estimate is limited: the true effect may be substantially different from the estimate of the effect.
**Very low certainty:** we have very little confidence in the effect estimate: the true effect is likely to be substantially different from the estimate of effect.

***The risk in the intervention group** (and its 95% confidence interval) is based on the assumed risk in the comparison group and the **relative effect** of the intervention (and its 95% CI).
**SE-SR:** self-expanding stent-retriever; **CI:** confidence interval; **OR:** odds ratio

a. No heterogeneity with consistent effect but only moderate certainty due to retrospective design.

b. Moderate heterogeneity (I2 >50-75%) and retrospective evidence limit certainty of evidence.

**Figures S1-S3. Risk of Bias for secondary outcomes**


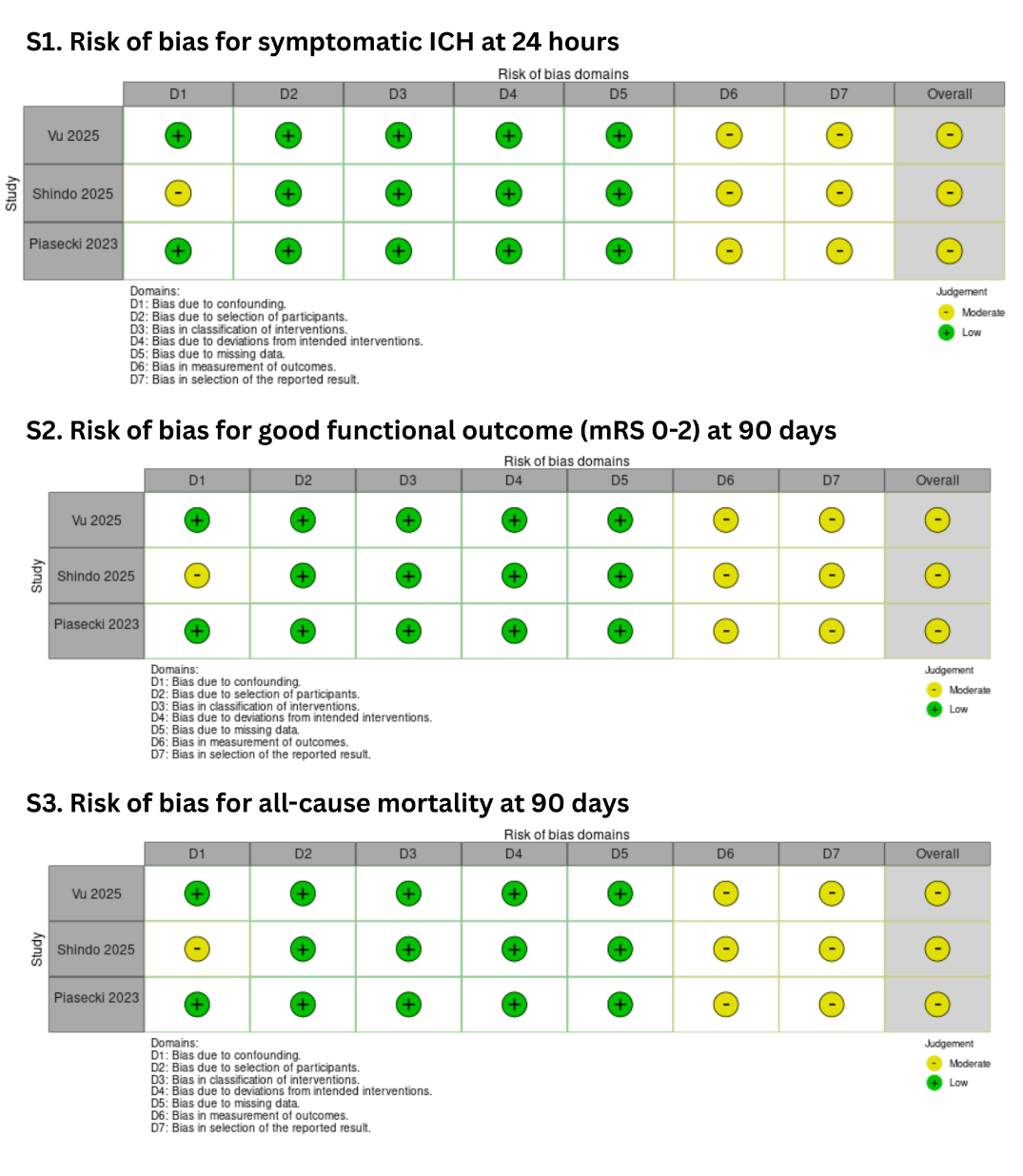

Supplement: Supplementary file 1 [file Supplementary_file_1.docx]
